# Effects of a music-accompanied multimodal exercise program on trunk endurance, stress, and anxiety in university students: a randomized controlled trial

**DOI:** 10.1590/1806-9282.20260773

**Published:** 2026-10-30

**Authors:** Emine Merve Ersever, Osman Şenol Yıldız, Emre Ersöz

**Affiliations:** 1 Ankara University, Haymana Vocational School, Department of Therapy and Rehabilitation, Physiotherapy Program – Ankara, Turkey.; 2 Etlik Public Hospitals Deputy Directorate, Department of Physical Medicine and Rehabilitation – Ankara, Turkey.

**Keywords:** Exercise therapy, Music therapy, Physical endurance, Stress, psychological, Anxiety, Students

## Abstract

**BACKGROUND:**

Multimodal interventions integrating breathing regulation and rhythmic auditory stimulation may improve trunk stability and modulate stress-related psychophysiological responses. Evidence on combined core stabilization and autonomic regulation programs in young adults remains limited.

**OBJECTIVE:**

The aim of this study was to investigate the effects of a 12-week music-accompanied program combining core stabilization, diaphragmatic breathing, and relaxation exercises on trunk muscle endurance and stress-related outcomes in university students.

**METHODS:**

In this randomized controlled trial, 80 university students were randomly assigned to an intervention (n=40) or a waitlist control group (n=40). The intervention was performed four times weekly for 12 weeks under physiotherapist supervision, including progressive core stabilization integrated with diaphragmatic breathing and slow-tempo music. Outcomes included trunk extensor endurance (Biering-Sorensen), lateral trunk endurance (lateral plank), trunk flexor endurance, perceived stress (PSS-10), and state anxiety (STAI-S). A 2×2 mixed-design analysis of variance evaluated group×time interactions. Assessors were not blinded.

**RESULTS:**

Significant group×time interactions favored the intervention group for perceived stress (η^2^p=0.148, d=0.82), state anxiety (η^2^p=0.122, d=0.74), lateral trunk endurance (left lateral plank: η^2^p=0.052, d=0.46;right lateral plank: η^2^p=0.078, d=0.57), and trunk flexor endurance (η^2^p=0.123, d=0.74). No significant interaction was found for trunk extensor endurance.

**CONCLUSION:**

A 12-week multimodal program combining core stabilization, breathing, relaxation, and music improved trunk endurance and reduced stress and anxiety; the waitlist design does not permit isolating music's specific contribution.

## INTRODUCTION

University students face increasing psychological and physical challenges due to academic pressures, social expectations, and uncertainty about the future. These stressors may manifest as mental fatigue, physical tension, postural dysfunction, and reduced physical endurance^1^. Holistic interventions addressing both psychological well-being and physical health are gaining attention, particularly those rooted in mindfulness and controlled movement^2^.

The core musculature plays a critical role in postural control, functional movement efficiency, and injury prevention^2^. In sedentary populations such as university students, core weakness often contributes to poor posture and reduced trunk endurance^3^. Mindfulness-based breathing and movement practices enhance physical control while improving recovery from physical and psychological stress^2^. Interventions that stimulate the parasympathetic nervous system are highly relevant in rehabilitation contexts, given that physical and psychological stress frequently manifest through sympathetic overactivation^4^.

The diaphragm forms the superior boundary of the core unit together with the pelvic floor, transversus abdominis, and multifidus. During diaphragmatic inspiration, rib-cage expansion raises intra-abdominal pressure and thoracolumbar fascial tension, providing anticipatory lumbar stabilization^5,6^. This biomechanical link is the rationale for training breathing and core stability together, rather than as separate components.

Slow-tempo music can improve breathing rhythm, promote relaxation, and enhance concentration, integrating a mind-body experience^7^. Progressive relaxation exercises following physical exertion help individuals recognize and release physical tension, restoring physiological balance^7,8^. Contemporary models of stress emphasize bidirectional interaction between physiological activation and cognitive appraisal processes. Slow, regular auditory rhythms can entrain breathing toward a slower pattern linked to greater vagal tone; combined with core training, this offers a plausible route by which a single multimodal program could act on both trunk endurance and stress reactivity.

Few studies have tested core stabilization, breathing, and music together in a single trial in university students. The objective of this study was to assess the effects of a holistic movement program integrating breathing exercises, trunk stabilization, and relaxation practices accompanied by slow-tempo music on stress, anxiety, and trunk muscle endurance in university students.

## METHODS

### Study design

A two-group, pretest–posttest randomized controlled trial was approved by Ankara University Health Sciences Scientific Research and Publication Ethics Committee (E-79176220-050-04-1961949) and conducted in accordance with the Declaration of Helsinki. The study is registered at ClinicalTrials.gov (NCT07250438) and adheres to CONSORT guidelines. Randomization used the sequentially numbered opaque sealed envelopes (SNOSE) method^9^, overseen by an independent researcher. The 12-week intervention incorporated breathing, core, and relaxation exercises accompanied by music; the waitlist control group received no intervention during the study period. After study completion, control-group participants who wished to do so were offered the opportunity to undergo the same exercise program, consistent with a waitlist-control design. Participants could not be blinded to allocation given the nature of the intervention, and outcome assessors were also not blinded to group allocation (see "Limitations").

### Participants

Eighty university students volunteered and were randomly assigned to the exercise group (n=40; 30 females, 10 males) or control group (n=40; 32 females, 8 males). Mean participant age was 21 years. One participant from the exercise group withdrew for personal reasons, yielding a final exercise group of n=39 for analysis (Figure 1).

**Figure 1:**
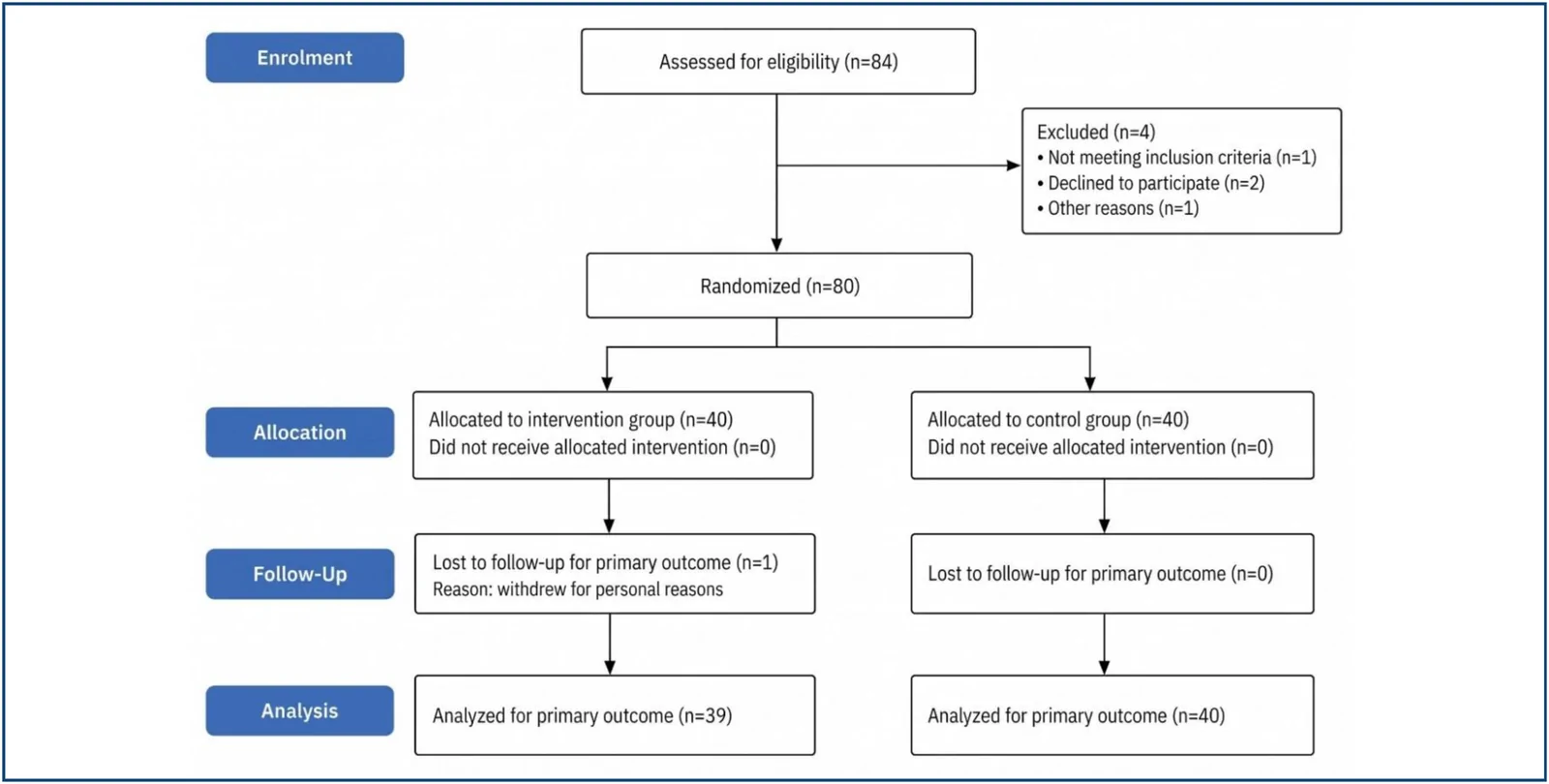
CONSORT flow diagram.

### Outcome measures

Measurements were collected at baseline and post-12-week intervention. Psychological outcomes included the Perceived Stress Scale (PSS-10; α=0.78)^10^ and the State-Trait Anxiety Inventory, State version (STAI-S; α: 0.86–0.95)^11^. Physical outcomes were assessed using the Biering-Sorensen Test for trunk extensor endurance (ICC>0.90)^12^, the Lateral Plank Test (bilateral), and the Trunk Flexor Endurance Test^13^.

### Intervention protocol

Sessions were conducted four times weekly for 12 weeks (≈45–60 min/session) under physiotherapist supervision. Each session comprised: (1) diaphragmatic and rhythmic breathing exercises (10–15 min) synchronized with slow-tempo classical music; (2) progressive core stabilization exercises (20–25 min) targeting abdominal, lumbar, and pelvic stabilizers, including planks, bridges, and trunk flexion/extension holds; and (3) progressive muscle relaxation and mindfulness-based stretching (10–15 min) with continued soft instrumental music.

Adherence was excellent: 35 of 39 participants attended all 48 scheduled sessions, and the remaining 4 used their full permitted 3-day absence allowance (45/48 sessions each), yielding a mean attendance of 47.7±0.9 of 48 sessions (99.4%); no participant was excluded from analysis for low attendance.

### Statistical analysis

Power analysis (G*Power 3.1) confirmed adequate power for medium-sized effects (paired t-tests: d=0.5, α=0.05, power≈0.80; 2×2 mixed analysis of variance (ANOVA): f=0.25, α=0.05, power: 0.82–0.87). Between-group comparisons used Student's unpaired t-test; within-group changes were evaluated using paired t-tests. A 2×2 mixed-design ANOVA assessed group×time interactions, with partial eta-squared (η^2^p) and Cohen's d reported as effect sizes. Statistical significance was set at p<0.05. This indicated ≥34 per group; 40 per group were recruited to allow for attrition.

## RESULTS

Eighty-four students were assessed for eligibility; 4 were excluded (1 not meeting inclusion criteria, 2 declined, 1 for other reasons), leaving 80 randomized. One exercise-group participant withdrew for personal reasons (final n=39 exercise, n=40 control). Baseline parameters were comparable between groups (Table 1).

**Table 1:** Baseline comparison between groups.

Parameter	Exercise (n=39)	Control (n=40)
Age (years)	21.15±1.60	20.02±1.29
BMI (kg/m^2^)	24.54±3.31	24.97±3.26
PSS (score)	21.58±3.84	20.64±3.38
STAI-S (score)	43.20±8.49	44.87±9.18
LLP (sec)	34.9±20.3	36.5±21.7
RLP (sec)	37.8±21.5	39.9±22.1
BS (sec)	93.8±43.9	84.7±36.1
TFE (sec)	73.9±45.6	70.8±33.4

Mean±SD, unpaired t-test, no significant differences. BMI: body mass index; PSS: Perceived Stress Scale; STAI-S: State-Trait Anxiety Inventory; LLP: left lateral plank; RLP: right lateral plank; BS: Biering-Sørensen; TFE: trunk flexor endurance.

Following 12 weeks, the exercise group improved significantly on all outcomes except trunk extensor endurance; BMI fell by 0.58 kg/m^2^ (p=0.003), not deemed clinically meaningful. Between-group change scores favored the exercise group for PSS, STAI-S, LLP, RLP, and TFE, with the largest effects for PSS (η^2^p=0.148, d=0.82) and STAI-S (η^2^p=0.122, d=0.74); BS showed no significant between-group difference (Table 2).

**Table 2:** Within-group change (exercise) and between-group comparison of change scores (Δ).

Parameter	Ex. baseline	Ex. Post	Δ Exercise	Δ Control
BMI (kg/m^2^)	24.54±3.31	23.96±3.26*	0.12±0.36	0.11±0.37
PSS	21.92±3.75	17.91±3.58*	6.8±7.2	1.4±5.9*
STAI-S	43.20±8.49	33.94±6.88*	8.9±9.5	2.3±8.4*
LLP (sec)	34.9±20.3	48.5±25.4*	13.6±17.9	5.7±16.2*
RLP (sec)	37.8±21.5	52.3±26.7*	14.5±18.5	4.7±15.6*
BS (sec)	93.8±43.9	107.95±49.7*	14.15±35.4	2.1±35.4
TFE (sec)	73.9±45.6	103.4±63.8*	29.5±40.3	3.2±30.1*

* p<0.05. Δ: post minus pre. Exercise n=39, Control n=40. Ex.: Exercise; BMI: body mass index; PSS: Perceived Stress Scale; STAI-S: State-Trait Anxiety Inventory; LLP: left lateral plank; RLP: right lateral plank; BS: Biering-Sørensen; TFE: trunk flexor endurance.

## DISCUSSION

This study investigated the effects of a multimodal program combining breathing, core stabilization, relaxation, and music on stress, anxiety, and trunk endurance in university students. The intervention produced significant reductions in perceived stress and state anxiety, alongside improvements in lateral trunk and trunk flexor endurance, with moderate-to-large effect sizes.

Core stabilization exercises improved lateral plank and trunk flexor endurance but not trunk extensor endurance, possibly reflecting the anterior/lateral emphasis of the exercises used relative to posterior extensors. These results align with previous findings that core stabilization promotes physical function and psychological well-being^9-11^. The connection between body awareness, postural control, and psychological resilience supports the use of such exercises in integrated rehabilitation strategies.

These findings are broadly consistent with previous multimodal interventions in non-clinical young-adult samples: mindfulness-based movement and breathing programs have reported reductions in perceived stress of comparable magnitude in university populations^2^, although few studies have simultaneously tracked trunk endurance outcomes. Core-only stabilization programs typically report gains confined to the specifically trained muscle groups^3^; the broader pattern observed here, spanning both physical and psychological domains, may reflect the added breathing and relaxation components rather than core training alone. The absence of a trunk extensor effect similarly mirrors reports that posterior-chain endurance adapts more slowly than anterior or lateral musculature within short-duration protocols^13^, suggesting that 12 weeks may be insufficient for full extensor adaptation even where anterior/lateral gains are already evident.

Music reduces anxiety and improves emotional regulation, and when combined with progressive relaxation, further amplifies stress reduction^12-14^. The psychological improvements are consistent with prior work on music-supported relaxation^12-14^, but because music, breathing, and core exercise were always delivered together, their individual contributions cannot be separated in this design; the reductions in PSS and STAI-S should be attributed to the combined program.

Slow-tempo music may facilitate parasympathetic activation and support emotional processing^15,16^. Diaphragmatic breathing has independently been identified as a technique for reducing psychological and physiological stress^17^. Its integration with rhythmic movement and auditory stimulation may create a synergistic effect on autonomic regulation, consistent with the observed reductions in PSS and STAI-S^17-19^.

The relaxation phase appeared to contribute to psychophysical recovery. During inspiration, thoracic extension and rib-cage elevation raise intra-abdominal pressure via lateral abdominal expansion, improving trunk stability through thoracolumbar fascial tension^5,6^. This suggests the combined core-and-breathing protocol contributed to both endurance gains and post-exercise relaxation. Beyond this passive biomechanical coupling, repeated diaphragmatic loading during core exercise may itself train respiratory musculature, raising the possibility of a bidirectional relationship in which improved breathing control supports trunk endurance and improved trunk control supports more efficient breathing. Disentangling this bidirectional relationship was beyond the scope of the outcome measures used here but represents a promising direction for future mechanistic studies incorporating respiratory function testing alongside core endurance assessment.

### Clinical relevance

The between-group change in PSS-10 (Δ6.8 vs. Δ1.4) and STAI-S (Δ8.9 vs. Δ2.3) exceeded the control group's change well beyond the minimal clinically important difference reported for the PSS-10 in a university student population (2.19–2.66 points)^20^, suggesting a real effect rather than noise. A supervised, low-cost, group-based program of this kind could be feasibly delivered through university health or physiotherapy services as a preventive option for students. Clinically important difference thresholds for the endurance tests used are not well established in healthy young adults, limiting clinical (as opposed to statistical) interpretation of the physical findings. A lower-frequency variant could be piloted to test dose-response relationships and improve feasibility for academic calendars.

### Limitations

Most importantly, the waitlist (rather than active) control does not account for non-specific effects of attention, supervision, or group participation, and prevents disentangling music, breathing, and core-training effects; conclusions about specific active ingredients remain preliminary. Outcome assessors were also not blinded, which may have biased the physical endurance tests. The sample was predominantly female (30/39 exercise, 32/40 control), limiting generalizability to male students. Future research should incorporate active comparison groups (exercise-only, music-only, and relaxation-only), assessor blinding, more balanced sex distribution, and extended follow-up to isolate therapeutic components.

## CONCLUSION

A 12-week multimodal program integrating core stabilization, diaphragmatic breathing, relaxation, and music was associated with clinically meaningful reductions in perceived stress and state anxiety, alongside improvements in lateral and anterior trunk endurance, in university students. These findings are consistent with evidence that combining breathing regulation with movement-based training can act on both biomechanical and parasympathetic pathways. Because the trial used a waitlist rather than an active control, the findings support the multimodal program as a whole; the specific contribution of music cannot be determined from this design. Larger, active-comparator trials with assessor blinding and objective physiological measures are warranted to disentangle individual components and confirm these effects.

## Data Availability

The datasets generated and/or analyzed during the current study are available from the corresponding author upon reasonable request.
